# A test of general relativity using the LARES and LAGEOS satellites and a GRACE Earth gravity model: Measurement of Earth’s dragging of inertial frames

**DOI:** 10.1140/epjc/s10052-016-3961-8

**Published:** 2016-03-04

**Authors:** Ignazio Ciufolini, Antonio Paolozzi, Erricos C. Pavlis, Rolf Koenig, John Ries, Vahe Gurzadyan, Richard Matzner, Roger Penrose, Giampiero Sindoni, Claudio Paris, Harutyun Khachatryan, Sergey Mirzoyan

**Affiliations:** 1 Dipartimento Ingegneria dell’Innovazione, Università del Salento, Lecce, Italy; 2 Museo della fisica e Centro studi e ricerche Enrico Fermi, Rome, Italy; 3 Scuola di Ingegneria Aerospaziale, Sapienza Università di Roma, Rome, Italy; 4 Joint Center for Earth Systems Technology (JCET), University of Maryland, Baltimore County, USA; 5 Helmholtz Centre Potsdam, GFZ German Research Centre for Geosciences, Potsdam, Germany; 6 Center for Space Research, University of Texas at Austin, Austin, USA; 7 Center for Cosmology and Astrophysics, Alikhanian National Laboratory and Yerevan State University, Yerevan, Armenia; 8 Theory Center, University of Texas at Austin, Austin, USA; 9 Mathematical Institute, University of Oxford, Oxford, UK; 10 DIAEE, Sapienza Università di Roma, Rome, Italy

## Abstract

We present a test of general relativity, the measurement of the Earth’s dragging of inertial frames. Our result is obtained using about 3.5 years of laser-ranged observations of the LARES, LAGEOS, and LAGEOS 2 laser-ranged satellites together with the Earth gravity field model GGM05S produced by the space geodesy mission GRACE. We measure $\mu = (0.994 \pm 0.002) \pm 0.05$, where $\mu$ is the Earth’s dragging of inertial frames normalized to its general relativity value, 0.002 is the 1-sigma formal error and 0.05 is our preliminary estimate of systematic error mainly due to the uncertainties in the Earth gravity model GGM05S. Our result is in agreement with the prediction of general relativity.

## Introduction

About 100 years ago Albert Einstein completed the publication of a series of fundamental papers describing the gravitational theory known as general relativity (GR) [1–7]. Since then Einstein’s gravitational theory has had experimental and theoretical triumphs, including the prediction and observation of the expansion of the universe, of black holes, gravitational lensing and gravitational waves [8–14]. GR has today a number of practical applications to our everyday life [15] including its corrections that enable the Global Navigation Satellite System to reach accuracies at the level of a few decimetres [16].

Nevertheless, GR has not been reconciled with the other fundamental theory of modern physics: quantum mechanics. Further, Einstein’s gravitational theory predicts the occurrence of spacetime singularities where every known physical theory ceases to be valid, the spacetime curvature diverges and time ends [17]. In 1998 observations of distant supernovae of type Ia implied the quite surprising result that the universe has an accelerated expansion [18, 19]. An explanation for this mysterious result can be found in the cosmological constant introduced by Einstein to avoid a dynamical universe and later, in 1931, abandoned by Einstein himself. However, the cosmological constant corresponds to vacuum energy and quantum field theory predicts that the vacuum energy should have a value approximately $10^{122}$ times larger than the *dark energy* [20, 21] density that is observed in the universe. To explain the accelerated expansion of the universe, dark energy should compose more than 70 % of our universe, but its real nature is unknown. Other explanations include a time dependent vacuum energy with the exotic name of quintessence, and modifications of GR such as the so-called f(R) theories. Therefore, in spite of its experimental triumphs, Einstein’s gravitational theory continues to need further accurate tests at all scales from solar system tests to astrophysical and cosmological observations.

Successful tests [11–13] of effects and phenomena predicted by GR include the well known perihelion precession of Mercury (and in general the periastron advance of an orbiting body), the equivalence principle and the time-dilation of clocks in a gravitational field, the deflection and time-delay of electromagnetic waves by a mass, the dynamics of the Moon, accurately measured by Lunar Laser Ranging(LLR) and of binary pulsars [22–24], gravitational lensing and other relevant astrophysical observations. Gravitational waves have been indirectly observed at the level predicted by GR from the rate of change of the orbital period of the binary pulsar PSR B1913 + 16 [22]. Recently the two LIGO advanced detectors (Caltech and MIT) have directly detected the gravitational waves from the inspiral and merger of a pair of black holes [14] marking the beginning of the gravitational-wave astronomy.

## Dragging of inertial frames

Among the intriguing phenomena predicted by GR, and so far only tested with approximately 10 % accuracy, is the “dragging of inertial frames”, or “frame-dragging” as Einstein named it in 1913 [25]. Frame-dragging has relevant astrophysical applications to the dynamics of matter falling into rotating black holes and of jets in active galactic nuclei and quasars [26].

A test-gyroscope is a small current of mass in a loop and may be realized using a sufficiently small spinning top. In GR a gyroscope determines the axes of local nonrotating inertial frames. In such frames the equivalence principle holds so that the gravitational field is locally unobservable and all the laws of physics are the laws of special relativity theory. However, in GR a gyroscope has a potential behavior different from that in classical Galilei–Newton mechanics. In classical mechanics, a torque-free gyroscope is predicted to always point toward the same distant “fixed” stars. In contrast, in GR, a gyroscope is dragged by mass currents, such as the spinning Earth, and therefore its orientation can change with respect to the distant “fixed” stars. If we were to rotate with respect to the gyroscope, we would feel centrifugal forces, even though we may not rotate at all with respect to distant “fixed” stars [11].

Frame-dragging of a gyroscope is formally similar to the change of orientation of a magnetic dipole by a magnetic field generated by an electric current in electrodynamics [26]. In GR, a current of mass generates an additional contribution to the gravitational field, called gravitomagnetic field because of its formal analogy with electrodynamics. The gravitomagnetic field then exerts a torque on a gyroscope in the same way a magnetic field torques a magnetic needle in electrodynamics.

In 1918, Lense and Thirring [27] published the equations of the frame-dragging perturbations of the orbital elements of a satellite in the weak gravitational field of a slowly rotating body. The rate of change of the nodal longitude of the satellite, known as the Lense–Thirring effect, is given by $\varvec{\dot{\Omega }} \, = \,{2 \mathbf{J} \over a^3 \, (1 - e^2)^{3/2}}$, where ${\varvec{\Omega }}$ is the nodal longitude of the satellite, *a* its semimajor axis, *e* its orbital eccentricity, and $\mathbf{J}$ is the angular momentum of the rotating body. We recall that the node, ascending or descending, of a satellite is defined as the intersection of its orbit with the equatorial plane of the central body, in our case the Earth [28].

Frame-dragging was observed [29] in 1997–1998 by using the LAGEOS (LAser GEOdynamics Satellite) and LAGEOS 2 laser-ranged satellites [30] and measured with approximately 10 % accuracy [31–34] in 2004–2010, using LAGEOS, LAGEOS 2 and the Earth’s gravity field determinations by the space geodesy mission GRACE [35, 36]. In 2011 the dedicated space mission Gravity Probe B, launched in 2004 by NASA, reported also a test of frame-dragging with approximately 20 % accuracy [37].

LAGEOS was launched in 1976 by NASA, and LAGEOS 2 in 1992 by ASI and NASA [30]. They are two almost identical passive satellites covered with 426 corner cube reflectors to reflect back the laser pulses emitted by the stations of the satellite laser ranging (SLR) network [38]. SLR allows measurement of the position of the LAGEOS satellite with an accuracy that can reach a few millimetres over a range of about 6000 km. The twin GRACE (Gravity Recovery and Climate Experiment) satellites were launched in 2002 by NASA and DLR (the German Aerospace Center). They are 200–250 km apart, in a near-polar orbit at an altitude of about 480 km. The GRACE space mission has allowed extremely accurate determinations of the Earth’s gravitational field and its temporal variations. For the main characteristics and orbital parameters of LARES, LAGEOS, LAGEOS 2 and GRACE, see Table 1.

**Table 1:** Main characteristics and orbital parameters of the satellites used in the LARES experiment

	LARES	LAGEOS	LAGEOS 2	GRACE
Semimajor axis (km)	7821	12270	12163	6856
Eccentricity	0.0008	0.0045	0.0135	0.005
Inclination	$69.5^\circ$	$109.84^\circ$	$52.64^\circ$	$89^\circ$
Launch date	13 Feb 2012	4 May 1976	22 Oct 1992	17 Mar 2002
Mass (kg)	386.8	406.965	405.38	432
Number of CCRs	92	426	426	4
Diameter (cm)	36.4	60	60	

The test of frame-dragging with the LAGEOS satellites was obtained by using the two observables quantities given by the two nodal rates of LAGEOS and LAGEOS 2 for the two main unknowns: the frame-dragging effect and the uncertainty in the Earth’s quadrupole moment, $J_2$ [39]. If the Earth’s gravitational potential is expanded in spherical harmonics, the even zonal harmonics are those of even degree and zero order. They represent the deviations from spherical symmetry of the gravitational potential of a body which are axially symmetric and which are also symmetric with respect to the equatorial plane of the body. The main secular drifts of the nodal longitude of a satellite are due to the Earth’s even zonal harmonics. In particular, the largest node shift is by far due to the even zonal of degree two, $J_2$, i.e. the Earth’s quadrupole moment [28]. To measure frame-dragging we either need to perfectly determine the Earth’s even zonal harmonics or devise a method to neutralize the propagation of their uncertainties in our measurement.

## LARES

LARES is a satellite of the Italian Space Agency (ASI) launched by the European Space Agency with the new launch vehicle VEGA (ESA-ASI-ELV-AVIO). It is a passive, spherical laser-ranged satellite (see Table 1). The LARES satellite was designed to approach as closely as possible an ideal test particle [40]. This goal was mainly achieved by adopting the following design requirements: (1) minimize the surface-to-mass ratio, (2) reduce the number of parts, (3) avoid any protruding component, (4) use a non-magnetic material and (5) avoid the painting of the satellite surface. The first requirement was implemented by using a tungsten alloy [41], the most dense material on Earth with an acceptable cost and good manufacturing characteristics. With a diameter of 36.4 cm and a total mass of 386.8 kg, the final mean density of the satellite is 15317 kg/m$^3$, which makes LARES the known orbiting object in the solar system with the highest mean density and the satellite with the lowest surface-to-mass ratio. The second requirement was achieved by building the satellite body out of one single piece of tungsten alloy, thus reducing thermal contact conductance and consequently the onset of thermal gradients. Temperature differences on the surface of the LAGEOS satellites produce in fact a tiny but not negligible perturbation: the thermal thrust [42]. To comply with the third requirement, the satellite interface with the separation system was limited only to four hemispherical cavities machined on the equator of the satellite. The fourth and fifth requirements were simply fulfilled by choosing a non-magnetic tungsten alloy, although with slightly lower density than a magnetic tungsten alloy, with a proper surface treatment and with no painting [43].

**Fig. 1:**
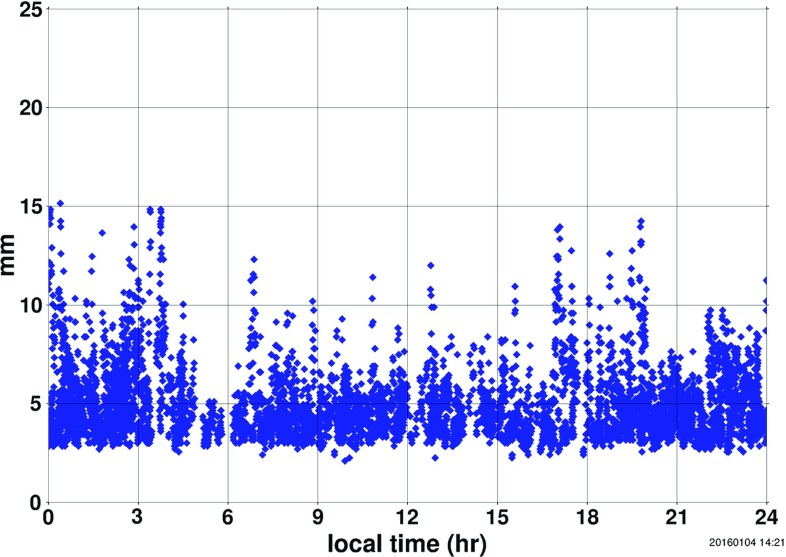
Root mean square (RMS) of the LARES normal points obtained from the laser ranging observations of the Graz station of the ILRS during 2015. The average RMS of the LARES normal points is 4.83 mm (courtesy of the ILRS [38])

## Test of frame-dragging using LARES and the two LAGEOS satellites

The basic idea of the LARES space mission is to couple its orbital data with those of the two LAGEOS satellites in order to have three observable quantities provided by the nodal rates of the three satellites [44]. The three observables can then be used to determine the three unknowns: frame-dragging and the two uncertainties in the two lowest degree even zonal harmonics, $J_2$, and $J_4$ (i.e. the spherical harmonics of degree 2 and 4 and order 0). In such a way the two largest sources of uncertainty in the nodal drift are eliminated, providing an accurate measurement of frame-dragging within our systematic uncertainty of a few percent.

Here we report on our orbital analysis of the laser ranging data of the LARES, LAGEOS, and LAGEOS 2 satellites from 26 February 2012 until 6 September 2015 using a prominent state-of-the-art Earth gravity field model, GGM05S [45]. GGM05S is an Earth gravity model released in 2013, based on approximately 10 years of GRACE data. It describes the Earth’s spherical harmonics up to degree 180. The laser ranging data of LARES, LAGEOS, and LAGEOS 2 were collected from more than 30 ILRS stations all over the world (see Fig. 1). We processed approximately 1 000 000 normal points of LARES, LAGEOS, and LAGEOS 2, corresponding to about 100 millions of laser ranging observations. The laser ranging normal points were processed using NASA’s orbital analysis and data reduction software GEODYN II [46], including the Earth gravity model GGM05S, Earth tides, solar radiation pressure, Earth albedo, thermal thrust, Lunar, solar and planetary perturbations, and the Earth rotation from Global Navigation Satellite System (GNSS) and Very Long Baseline Interferometry (VLBI).

The orbital residuals of a satellite are obtained by subtracting the observed orbital elements of the satellite with the computed ones. They provide a measurement of the orbital perturbations that, in the data reduction, are not included (un-modeled) or are modeled with some errors (mis-modeled) [29]. In particular, the residuals of the satellite’s node are due to the errors in the Earth’s even zonal harmonics and to the Lense–Thirring effect which we have not included in GEODYN II’s modeling. The Lense–Thirring nodal shift, theoretically predicted by general relativity, is about 30.7 milliarcsec/year on LAGEOS, about 31.5 milliarcsec/year on LAGEOS 2 and about 118.4 milliarcsec/year on LARES, the latter corresponding at the altitude of LARES to about 4.5 m/year.

**Fig. 2:**
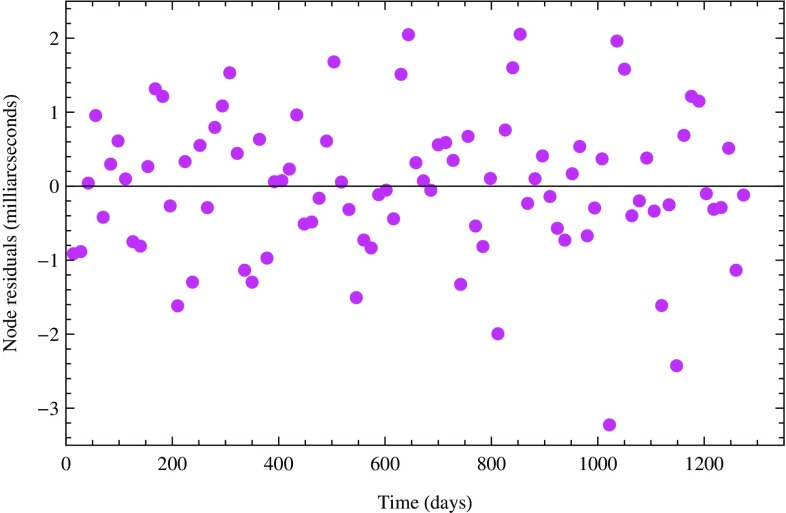
Combined residuals of LARES, LAGEOS, and LAGEOS 2, over about 3.5 years of orbital observations, after the removal of six tidal signals and a constant trend

Using the three observables provided by the three nodal rates of LAGEOS, LAGEOS 2 and LARES, we were able to eliminate not only the uncertainties in their nodal rates due to the errors in the even zonal harmonics $J_2$ and $J_4$ of the GGM05S model but also the uncertainties in their nodal rates due to the long and medium period tides contributing to the harmonics $J_2$ and $J_4$.

We fitted for the six largest tidal signals of LAGEOS, LAGEOS 2, and LARES, and for a secular trend, which produced

1 $$\begin{aligned} \mu = (0.994 \pm 0.002) \pm 0.05 \end{aligned}$$

Here $\mu$ = 1 is the value of frame-dragging normalized to its GR value, 0.002 is the formal 1-sigma error (the post-fit residuals of Fig. 2 show a normal–Gaussian– distribution to good approximation) and 0.05 is our conservative current estimate of systematic error due to the uncertainties in the Earth gravity field model GGM05S and to the other error sources. We discuss systematic errors below.

**Fig. 3:**
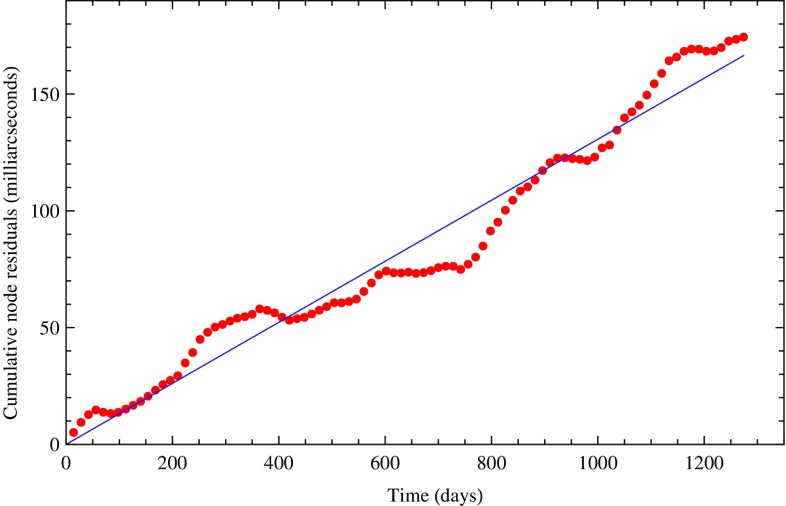
Fit of the cumulative combined nodal residuals of LARES, LAGEOS, and LAGEOS 2 with a linear regression only

**Fig. 4:**
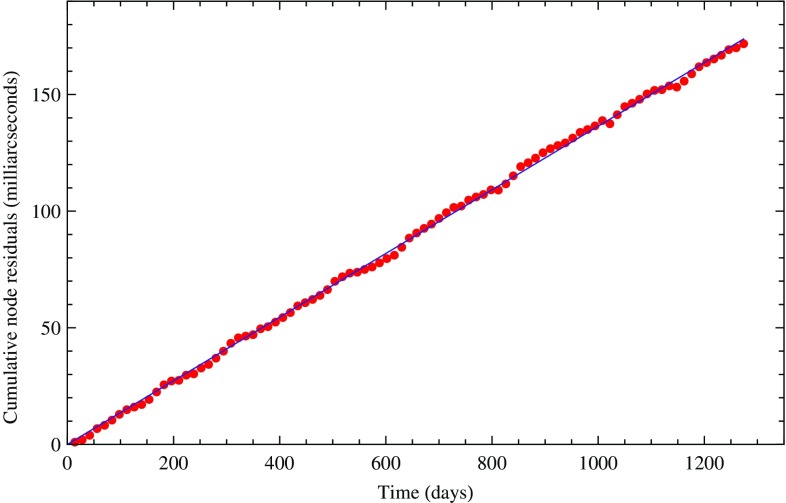
Fit of the cumulative combined nodal residuals of LARES, LAGEOS, and LAGEOS 2 with a linear regression plus six periodical terms corresponding to six main tidal perturbations observed in the orbital residuals

In Fig. 3, we display the least squares secular trend fit of the cumulative combined residuals of LAGEOS, LAGEOS 2 and LARES prior to fitting for the tides. In contrast, in Fig. 4 we show the secular trend obtained when including the six known periodical terms corresponding to the largest tidal signals observed on the satellite’s nodes. The fit is obviously much tighter. These tidal signals were identified both by a Fourier analysis of the observed residuals and by analytical computations of the main tidal perturbations of the nodes of the satellites. Some of the signals observed in the nodal residuals correspond to the perturbations due to the main non-gravitational perturbations.

The systematic errors in our measurement of frame-dragging with LARES, LAGEOS, and LAGEOS 2 are mainly due to the errors in the even zonal harmonics of GGM05S, used in our orbital fits with GEODYN II, with degree strictly larger than four. To evaluate these systematic errors, we tripled the published calibrated errors (i.e. including both the statistical and the systematic errors) of each even zonal coefficient of GGM05S (to multiply by a factor two or three is a standard technique in space geodesy to place an upper bound to the real error in the Earth’s spherical harmonics) and then propagated these tripled errors into the nodes of LARES, LAGEOS, and LAGEOS 2. We then found a systematic error of about 4 % in our measurement of frame-dragging due to the Earth’s even zonals.

Other smaller systematic errors are due to those long and medium period tides and non-gravitational perturbations either mis-modeled, or un-modeled. However, in our analysis we included the main tidal and non-gravitational perturbations, such as the direct radiation pressure from the Sun and the Earth, i.e. the albedo. Furthermore, the systematic errors due to the un-modeled or mis-modeled tidal and non-gravitational perturbations are periodical and their residual effect is quite small as clearly shown in the Fourier analysis of the post-fit orbital residuals shown in Fig. 2. Previous error analyses [32, 47–58] have confirmed that the systematic error due to tides, non-gravitational perturbations and other error sources is at the level of approximately 3 % and therefore the total root sum squared (RSS) systematic error, including the systematic error due to the Earth’s even zonals, is approximately at the level of 5 % if the LARES, LAGEOS, and LAGEOS 2 observations are used together with the Earth gravity field model GGM05S.

Although we are quite pleased with the analysis to date of frame dragging including LARES, LAGEOS, and LAGEOS 2, we consider this result only intermediate to a final determination. Our final result will present a careful restudy of systematics. We have been conservative here in quoting a 5 % estimate of our systematic error. Extending the observation time of LARES and the other satellites will improve our understanding of tidal contributions and will reduce the systematic error from that source. Different Earth gravity models lead to slightly different results, as is also the case for different orbital solvers. Completing a suite of solutions with different (up to date) Earth gravity models and different solvers will provide another estimate of the systematics. All these questions will be addressed in a forthcoming analysis of the measurement of frame-dragging using LARES, LAGEOS, LAGEOS 2 and GRACE.

However, we must also point out that the satellites LAGEOS, LAGEOS 2, and LARES will have tens of thousands of years on orbit, and they will remain useful to laser-ranged science for an extremely long time. Eventually the retroreflectors may become degraded, but LAGEOS has shown no sign of this in its 40 years on orbit. Other laser-ranged satellites will be launched to join the current ones. All these satellites will be available while at the same time better Earth gravity models, better orbital solvers, and better models of non-gravitational forces become available. The strength of this approach and these satellites is that they are available for innovative improvements in technique into the future.

## Conclusions

Using the laser-ranged satellites LARES, LAGEOS, and LAGEOS 2, and the Earth gravity field described by the GGM05S model based on GRACE observations, we obtained a test of frame-dragging: $\mu = (0.994 \pm 0.002) \pm 0.05$, where $\mu = 1$ is the theoretical prediction of general relativity, 0.002 is the 1-sigma statistical error and 0.05 is a conservative preliminary estimate of systematic error due to the uncertainties in the Earth gravity field model GGM05S and other error sources.
